# No association between pyrite content and lung cell responses to coal particles

**DOI:** 10.1038/s41598-021-87517-z

**Published:** 2021-04-14

**Authors:** Graeme R. Zosky, Ellen J. Bennett, Macarena Pavez, B. Basil Beamish

**Affiliations:** 1 Menzies Institute for Medical Research, College of Health and Medicine, University of Tasmania, Hobart, TAS Australia; 2 Tasmanian School of Medicine, College of Health and Medicine, University of Tasmania, Hobart, TAS Australia; 3 Division of Health Sciences, University of Otago, Otago, New Zealand; 4 B3 Mining Services Pty Ltd, Brisbane, Australia; 5 School of Minerals and Energy Resources Engineering, University of New South Wales, Sydney, Australia

**Keywords:** Respiratory tract diseases, Risk factors

## Abstract

There has been an increase in the identification of cases of coal workers’ pneumoconiosis (CWP) in recent years around the world. While there are a range of possible explanations for this, studies have implicated the pyrite content of coal as a key determinant of CWP risk. However, experimental studies to support this link are limited. The aim of this study was to assess the association between the pyrite content, and subsequent release of bioavailable iron, in coal particles and the response of lung cells involved in the pathogenesis of CWP (epithelial cells, macrophages and fibroblasts). Using real-world Australian coal samples, we found no evidence of an association between the pyrite content of the coal and the magnitude of the detrimental cell response. We did find evidence of an increase in IL-8 production by epithelial cells with increasing bioavailable iron (p = 0.01), however, this was not linked to the pyrite content of the coal (p = 0.75) and we did not see any evidence of a positive association in the other cell types. Given the lack of association between the pyrite content of real-world coal particles and lung cell cytotoxicity (epithelial cells and macrophages), inflammatory cytokine production (epithelial cells, macrophages and fibroblasts), and cell proliferation (fibroblasts) our data do not support the use of coal pyrite content as a predictor of CWP risk.

## Introduction

Coal Workers’ Pneumoconiosis (CWP) is an incurable interstitial lung disease that results from chronic inhalation of respirable coal dust particles. In recent years there has been an increase in the reported cases of CWP around the world including in regions of the United States^1^ and, more recently, in Australia^2^. As a result, there has been a surge in interest in the potential contributors to the risk of developing CWP.

Many studies have shown a relationship between the chemistry of respirable particulate matter (PM) and the magnitude of the adverse respiratory response in a range of contexts^3,4^. In the case of coal derived PM, some studies have suggested that the pyrite content of coal is linked to the risk of CWP. For example, a study combining CWP data and coal quality information from the U.S. Geological Survey showed a correlation between the prevalence of CWP and the pyritic sulphur content of the coal^5^. Using a chemical modelling approach, this association was linked to the liberation of bioavailable iron by the pyrite^5^. This is a biologically plausible link, given the importance of iron metabolism in the regulation of cellular inflammation and tissue injury in the lung^6^.

However, few experimental studies have been conducted to assess this link directly in lung cells. One early study showed that the pyrite content of coal was associated with the liberation of reactive oxygen species, however the particles used were much larger than the respirable fraction and the effect on RNA degradation was characterised in yeast rather than lung cells^7^. A more recent study found that inflammation induced by coal particles in alveolar epithelial cells (A549) was greatest in particles with a high pyrite content, however the dose–response was not linear and other markers, such as generation of reactive oxygen species (ROS) and cytotoxicity, were higher in the samples without pyrite than those with relatively high levels of pyrite^8^.

While the development of CWP is clearly linked to chronic inhalation of coal particles, the pathogenesis of the disease is complex and involves multiple cell types within the lung^9,10^. CWP develops as a result of chronic inflammation due to long-term inhalation of coal particles. Persistent inflammation, which is initiated and driven by the response of epithelial cells and resident macrophages to the coal particles^8,11^, leads to tissue injury and a cycle of aberrant repair which promotes fibrosis and irreversible lung damage^12^. Thus, the respiratory response to coal inhalation is influenced by epithelial cells, as the first point of contact for inhaled particles, macrophages, which phagocytose the particles, and fibroblasts, which can drive the irreversible fibrosis that is characteristic of the disease^10^.

The overall aim of this study was to determine the association between the pyrite content of coal particles and the lung cell response, to further assess the reported link between pyritic coal and the risk of CWP. To achieve this, we assessed the effect of coal particles with different levels of pyrite on the cytotoxic and inflammatory response in epithelial cells and macrophages and the proliferative response in fibroblasts. We then assessed the association between these responses and bioavailable iron release in simulated lung fluids.

## Results

### Coal characteristics

#### Physical and chemical properties

With the exception of sample J, which consisted of concentrated pyrite associated with a non-coal band of the coal seam, all samples were highly volatile bituminous coals with a range of pyrite contents (Table 1). The particle size range was relatively consistent between samples (median: 3.52–6.57 µm; Table 1) and endotoxin levels were all below the limit of detection of the assay (0.1 EU/mL) (*data not shown*).

**Table 1:** Summary of the characteristics of the 10 coal samples used to assess the association between the pyrite content, iron release and the cellular response.

Sample	Pyrite (%)	Particle size (median—μm)
A	2.79	3.86
B	10.35	4.59
C	1.10	4.39
D	0.79	4.91
E	0.37	3.98
F	2.43	4.67
G	7.32	4.14
H	5.81	3.52
I	34.99	6.57
J	49.70	4.04

#### Iron bioavailability

**Figure 1:**
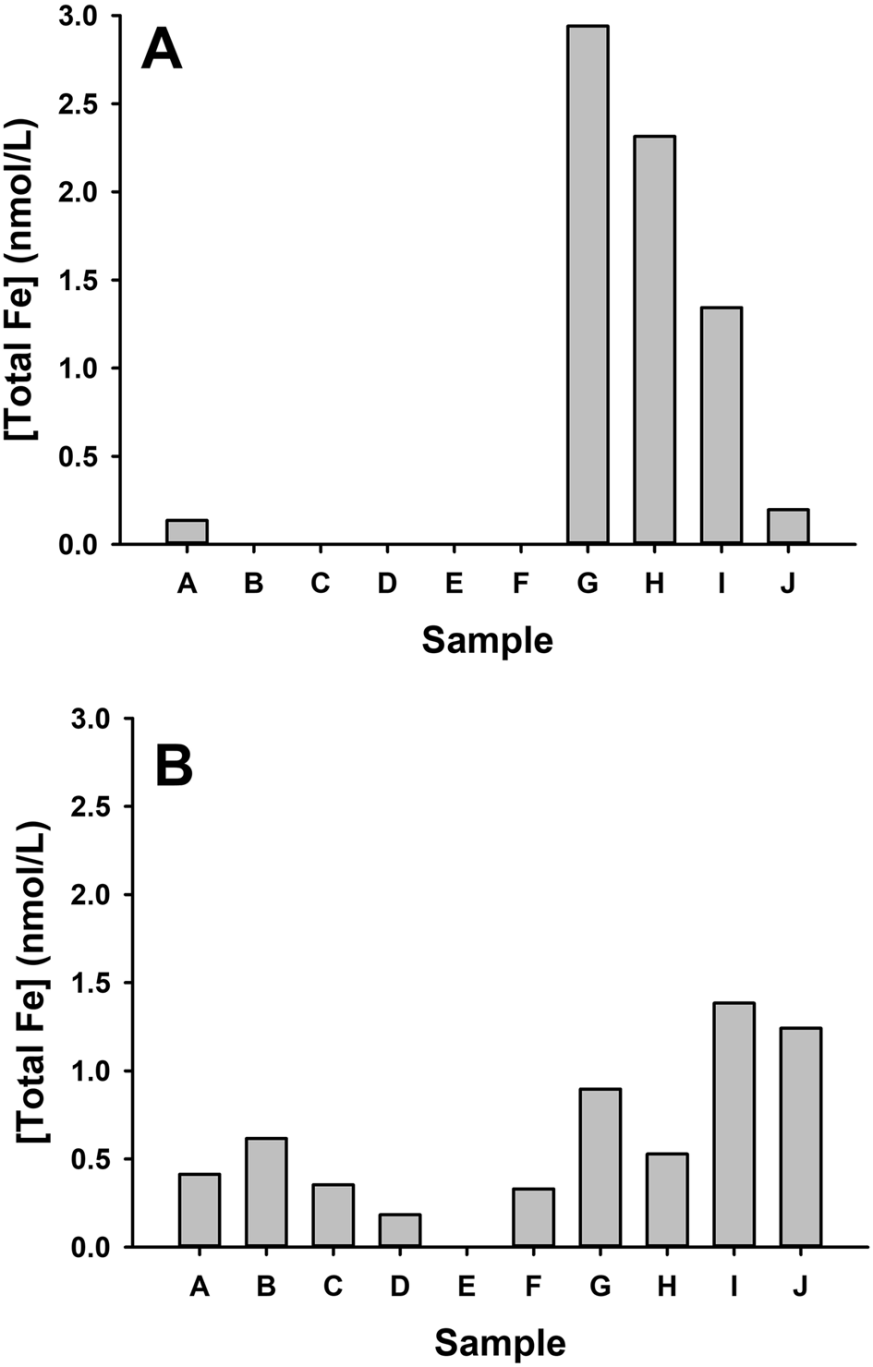
Bioavailable iron (total Fe) for the 10 coal samples measured after 24 h in Gamble’s solution (**A**) or artificial lysosomal fluid (ALF; **B**) measured by ferritin assay.

Iron release varied considerably between samples depending on which solution was used. The total iron content in the Gamble’s solution was far more variable than iron release into the ALF (Fig. 1); in almost all cases the release of iron was almost exclusively in the form of Fe^3+^ with relatively low levels of Fe^2+^ detected. There was no association between the pyrite content of the sample (R = 0.07; p = 0.85) and total iron release in Gamble’s solution. There was, however, a strong positive association between the pyrite content of the sample (R = 0.88; p < 0.001) and the total iron release in ALF.

### Epithelial cells

**Figure 2:**
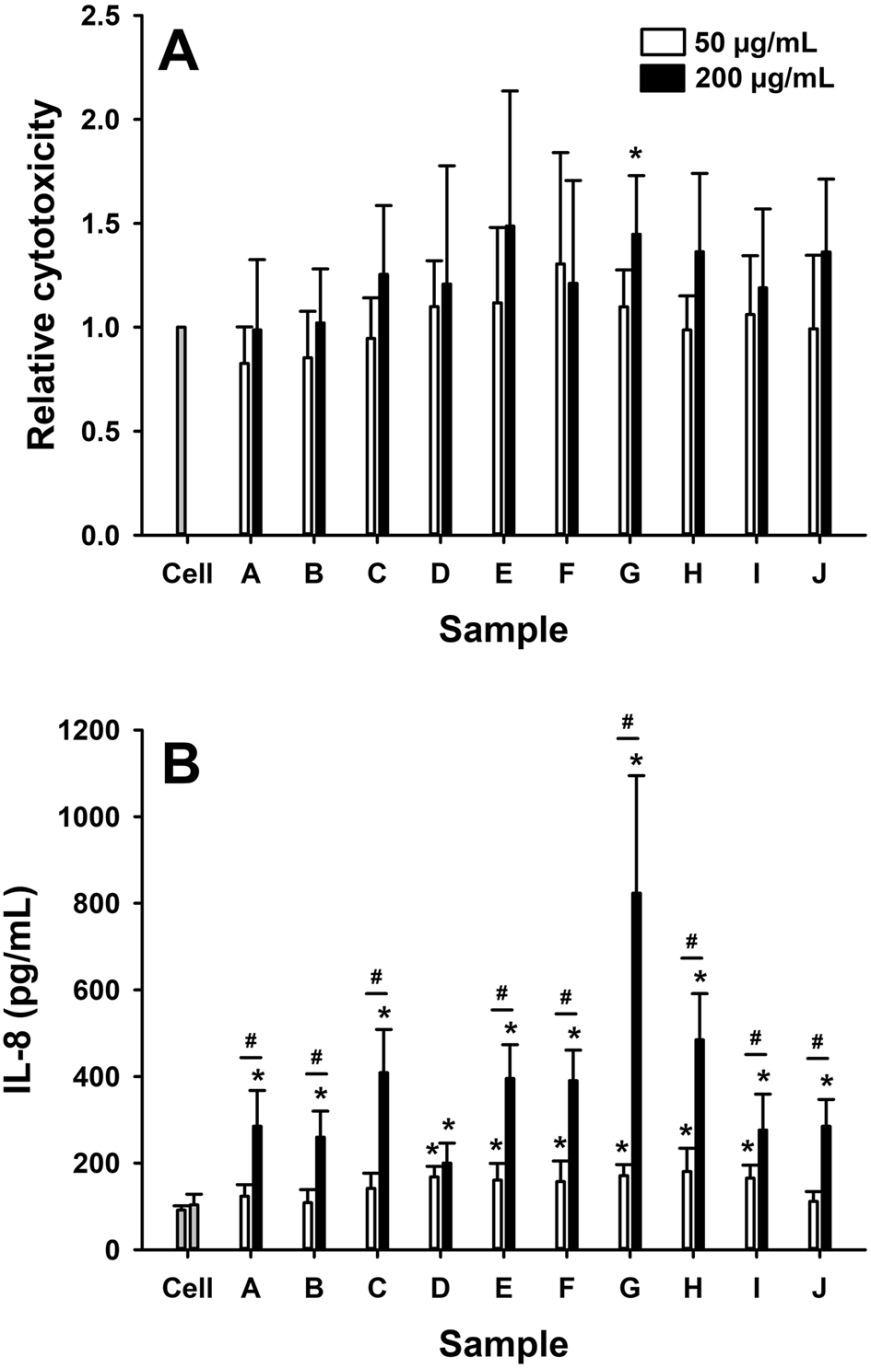
Relative cytotoxicity (**A**), measured as LDH release relative to control (cell only), and IL-8 production, measured in the cell supernatant by ELISA, in epithelial cells (A549) in response to 24 h exposure to 50 µg/mL (white bars) or 200 µg/mL (black bars) of coal particles (samples A–J). Data are presented as mean (SD). *p < 0.05, versus cell only; ^#^p < 0.05, 200 µg/mL vs 50 µg/mL.

Exposure to coal particles had minimal effect on cytotoxicity (LDH release) in A549 cells. While the response was variable, only the 200 µg/mL dose of sample G caused a statistically significant increase in LDH production (p = 0.04) compared to control (Fig. 2A). Samples D, E, F, G, H and I (p < 0.1 for all comparisons) caused an increase in IL-8 production by A549 cells at the 50 µg/mL dose compared to control levels (Fig. 2B). An increase in IL-8 production was detected in all samples at the 200 µg/mL (p < 0.001 for all comparisons versus control) which was dose-dependent in all coal samples except sample D (p = 0.22) (Fig. 2B). IL-6 production by A549 cells in response to the coal particles was also assessed, however, levels of this cytokine were below the limit of detection in all experiments (*data not shown*).

### Macrophages

**Figure 3:**
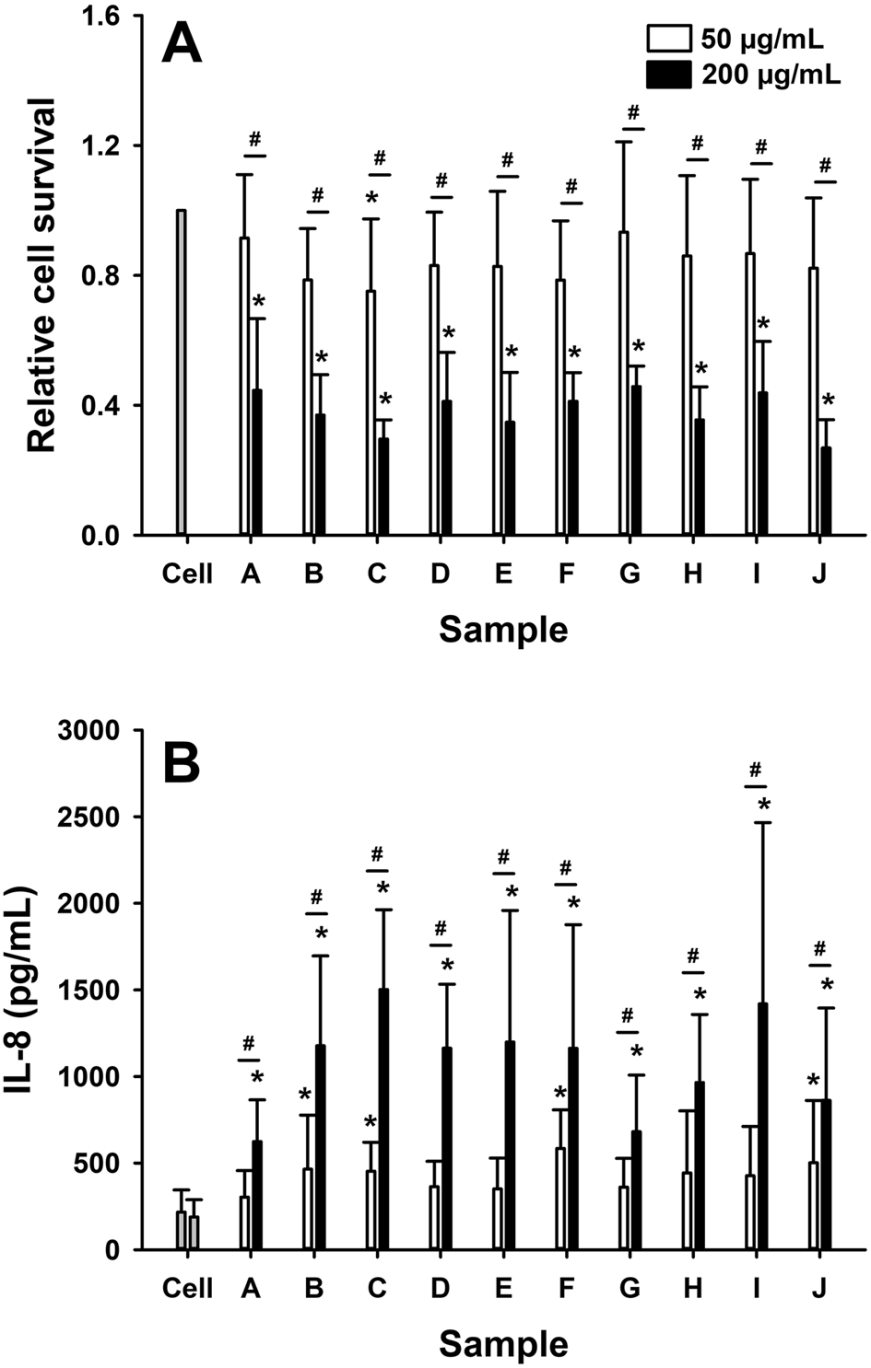
Relative cytotoxicity (**A**), measured by Trypan blue exclusion relative to control (cell only), and IL-8 production, measured in the cell supernatant by ELISA, in macrophages (THP-1 derived) in response to 24 h exposure to 50 µg/mL (white bars) or 200 µg/mL (black bars) of coal particles (samples A–J). Data are presented as mean (SD). *p < 0.05, versus cell only; ^#^p < 0.05, 200 µg/mL vs 50 µg/mL.

Exposure to coal particles caused dose-dependent decreases (p < 0.001 for 50 vs 200 µg/mL for all comparisons) in THP-1 cell survival (Fig. 3A). With the exception of sample C, where exposure to 50 µg/mL of particles caused a significant decrease in cell survival (p = 0.020), this cytotoxic effect was only detectable at the 200 µg/mL dose. In line with this response, exposure to all coal samples caused a dose-dependent (p < 0.001 for 50 vs 200 µg/mL for all comparisons) increase in IL-8 production in THP-1 cells (Fig. 3B). This effect was detectable at the 50 µg/mL dose for samples B, C, F and J (p < 0.02 for all comparisons) compared to control levels and for all coal samples at the 200 µg/mL dose (p < 0.001 for all comparisons). TNF-α and IL-1β production by THP-1 cells in response to the coal particles were also assessed, however, levels of these cytokines were below the limit of detection in all experiments (*data not shown*).

### Fibroblasts

**Figure 4:**
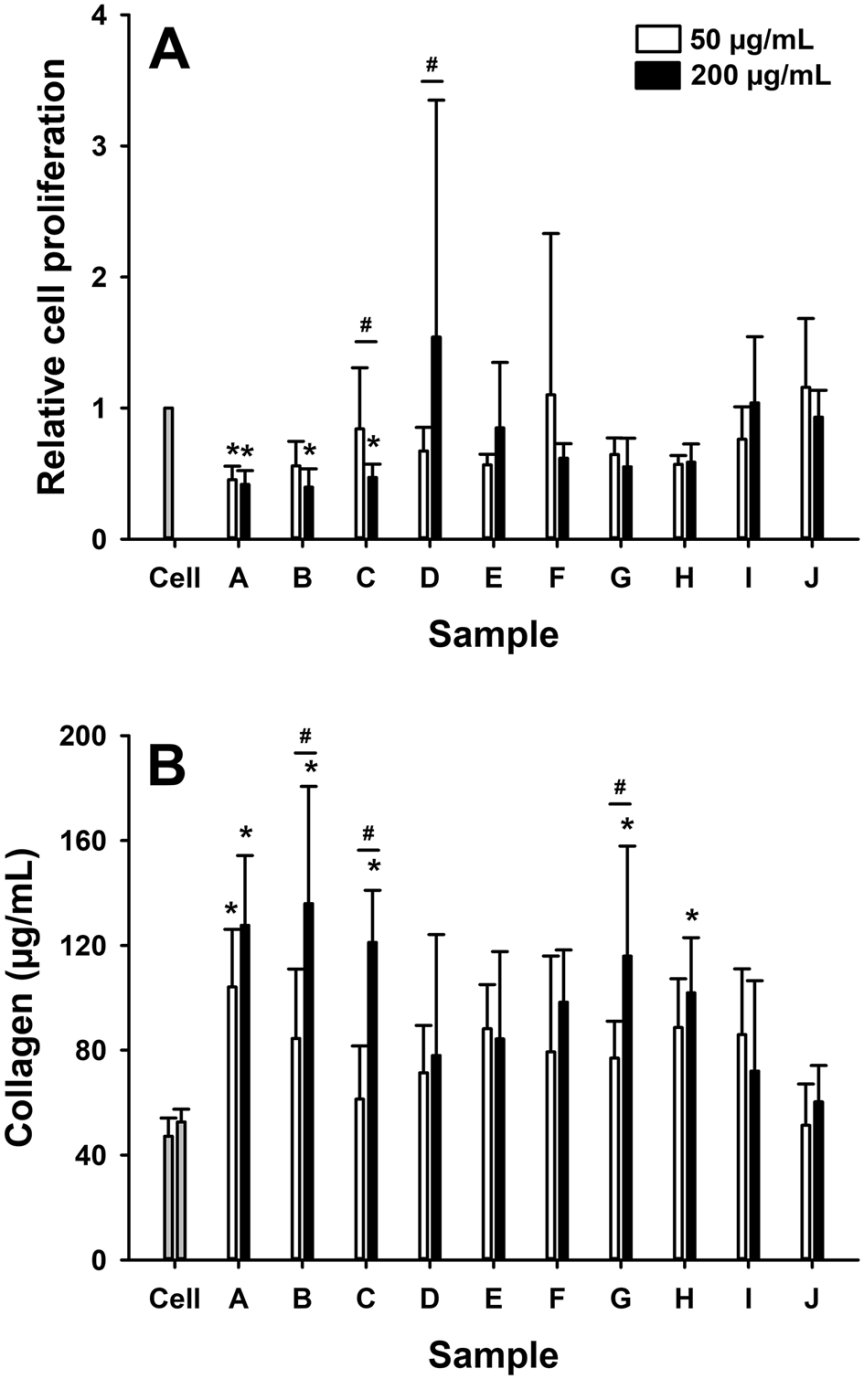
Relative cell proliferation (**A**), measured by WST-assay relative to control (cell only), and soluble collagen production, measured in the cell supernatant by colorimetric assay, in fibroblasts (CRL-1490) in response to 24 h exposure to 50 µg/mL (white bars) or 200 µg/mL (black bars) of coal particles (samples A–J). Data are presented as mean (SD). *p < 0.05, versus cell only; ^#^p < 0.05, 200 µg/mL vs 50 µg/mL.

The fibroblast proliferative response to exposure to coal particles was highly variable. Sample E, F, G, H, I and J had no effect on cell proliferation (p > 0.05 for all comparisons). In contrast, samples A (p < 0.01), B (p < 0.001) and C (p = 0.02) caused significant decreases in cell proliferation at the 200 µg/mL which could also be detected at 50 µg/mL for the A sample; implying these coal samples were cytotoxic to fibroblasts (Fig. 4A). In line with their adverse effect on proliferation, samples A (50 µg/mL, p < 0.01; 200 µg/mL, p < 0.001), B (200 µg/mL, p < 0.001) and C (200 µg/mL, p < 0.001) caused a significant increase in collagen production in fibroblasts compared to controls. In addition, exposure to 200 µg/mL of samples G (p = 0.001) and H (p = 0.03) also increased collagen production when fibroblasts were exposed to 200 µg/mL of these samples (Fig. 4B).

### Associations between the cellular response and the coal characteristics

Associations between the properties of the coal particles, with a focus on pyrite content and iron release (as appropriate to the cells used), and the cellular response to exposure to 200 µg/mL of particles were assessed by linear regression with adjustment for particle size as a covariate; outcomes of these analyses are summarised in Table 2. In summary, there was no association between the pyrite content of the coal and any of the measures of cytotoxicity, cell proliferation, cytokine or collagen production in any of the cell types (p > 0.1 for all comparisons). There was a significant association between iron release by the coal particles and IL-8 production in epithelial cells (p = 0.01), but no associations with any of the other outcome measure across cell types (p > 0.15 for all comparisons).

**Table 2:** Outcomes of linear regression analyses, adjusted for particle size, for the association between cell responses and the properties of the coal particles the cells were exposed to (pyrite content and bioavailable iron).

Cell type	Outcome	Pyrite		Bioavailable Iron	
		β [95% CI]	p	β [95% CI]	p
Epithelial cells	Cytotoxicity	0 [− 0.01, 0.01]	0.61	0.06 [− 0.05, 0.18]	0.24
	IL-8	− 1.31 [− 10.66, 8.03]	0.75	121.03 [40.49, 201.62]	**0.01**
Macrophages	Cytotoxicity	0 [0, 0]	0.18	− 0.02 [− 0.16, 0.17]	0.67
	IL-8	− 4.64 [− 17.24, 7.97]	0.41	− 299.78 [− 747.81, 148.24]	0.16
Fibroblasts	Proliferation	0 [− 0.02, 0.02]	0.78	− 0.04 [− 0.30, 0.22]	0.74
	Collagen	− 0.83 [− 1.96, 0.30]	0.13	0.71 [− 18.68, 20.09]	0.93

Bold indicates *p* < 0.05

## Discussion

The aim of this study was to determine whether there is an association between pyrite, the subsequent release of iron, and the cellular response to coal particles. We assessed this using real-world coal samples with a range of pyrite contents in key cell types that are involved in the pathogenesis of CWP. It was clear from our data that the magnitude of the cell response varied considerably between samples depending on the cell type and the outcome measure; suggesting that coal properties are likely to influence the risk of CWP. However, based on the cell-types we studied, we found no evidence of an association between the pyrite content of the coal and the magnitude of the detrimental cell response. We did find evidence of an increase in IL-8 production by epithelial cells with increasing bioavailable iron, however, this was not linked to the pyrite content of the coal and we did not see any evidence of an association in the other cell types. Collectively, these observations are contrary to the suggestion that the pyrite content of coal dust is a key determinant of CWP risk.

Our data clearly showed that coal particles were cytotoxic^10,13^, pro-inflammatory^10,14^ and pro-fibrotic^10^. This is consistent with the long history of literature on the health effects of coal dust; particularly chronic respiratory diseases including pneumoconiosis^15^. We observed substantial increases in production of IL-8 by epithelial cells and macrophages in our system. IL-8 is a pro-inflammatory cytokine and neutrophil chemo-attractant, that is known to be produced by epithelial cells and macrophages in response to a range of respiratory insults (pathogenic and non-pathogenic)^16^ and is a biomarker of the presence and severity of CWP^17^. The coal particles also induced significant cytotoxicity, particularly in macrophages, which is consistent with the role of tissue injury in CWP^18^. Similarly, exposure to coal particles caused a significant increase in the production of soluble collagen by fibroblasts; consistent with the fibrotic pathology of CWP^19^. Thus, the cell lines in our system had responses that are aligned with the known effects of coal particles on lung cells and the development of CWP.

Importantly, the magnitude of the responses outlined above varied considerably between coal samples. Given the relative consistency of the particle size between samples, likely due to the milling process, this observation suggests coal chemistry is an important determinant of the cell response and, hence, the CWP risk. The potential modifying effect of particle chemistry is well documented in the literature in a range of contexts related to coal dust^20,21^; however, contrary to some reports^7,8^, we found no evidence for an association between the pyrite content of the coal and its cytotoxic, inflammatory or fibrotic potential. It is worth noting that we do not have measures of other chemical components of the particles, as our focus was purely on pyrite as a determinant of CWP risk. However, we directly assessed the ability of pyrite to release free iron in biologically relevant simulated lung fluids. Consistent with the importance of pH in the release of iron by pyrite^22^ we found a strong positive association between the pyrite content of the coal and release of iron in the lower pH artificial lysosomal fluid (ALF), but not the simulated airway lining fluid. We found no evidence for an association between the release of iron in ALF, or pyrite, and the macrophage response. Given the key role that macrophages play in the pathogenesis of CWP^23^, this does not support a role for pyrite and/or iron availability in macrophage driven CWP pathology. Similarly, we found no association between pyrite content or iron release in simulated lung fluid and the proliferative potential or collagen production by fibroblasts—also suggest that the pyrite content, and iron release, by coal particles has no direct influence on fibrotic response by fibroblasts.

The seminal paper by Huang and colleagues^5^ showed a correlation between the prevalence of CWP and pyritic sulphur content of the coal seam in the mine location. This analysis was extended to estimate bioavailable iron release, based on modelled chemical interactions in the coal, which showed a strong correlation with CWP risk. This built on previous work showing a positive association between low molecular weight iron and lipid peroxidation in lung cells^24^ and follow up studies showing an association between the pyrite content of coal and the inflammatory response^8^. While it is worth noting that we found no association between the pyrite content of our coal samples and the outcomes in A549 cells, we did find a positive correlation with iron release in simulated lung fluid which is consistent with these observations. It is possible that there are other components of the coal that may have contributed to this correlation, for example in previous work showing an association between the pyrite content of coal particles and the inflammatory response in A549 cells, the samples with the highest pyrite/iron also had much higher mercury content than the low pyrite/iron samples^8^. This may be an issue given mercury is known to induce inflammation which casts some doubt on the strength of this association^25^. Nonetheless, the consistency of these observations suggested that iron release by the particles is indeed a determinant of the inflammatory response in this cell line; however, this is not necessarily directly correlated with the pyrite content as shown in our data.

Our study had several limitations which are worth noting. Firstly, we used immortalised cell lines each studied in isolation. While this could be improved through the use of co-exposure model, in vivo models and/or the use of the primary cells lines, this approach allowed us to screen a large number of coal samples in a range of cell types and was appropriate for the proof-of-principle nature of the study. In addition, the doses chosen are likely to be higher than real-world exposure on a particle per surface area basis, however, they are entirely consistent with similar toxicological studies and allowed us to test the hypothesis.

In summary, our study clearly showed, using real-world coal samples, that there is no association between the pyrite content of coal and the magnitude of the response in a range of important lung cell types that are relevant to the pathogenesis of CWP. While this does not take into account the impact of other aspects of coal chemistry on the potential release of bioavailable iron, it is an important observation as the regulatory assessment of the CWP hazard in the mining environment often considers the pyrite content of the coal.

## Methods

### Coal samples

10 coal samples (A–J) were sourced from Australian underground coal mine core samples and crushed. The pyrite content was calculated according to industry standards (AS1308.11-2002; R2013) by calculating the total iron content following dissolution in nitric acid and subtracting this quantity from the iron content of the sulphur following barium sulphate precipitation. Particle size was determine using scanning electron microscopy (Hitachi SU-70 at 1.5 kV). The diameter of all particles on 3 fields of view at 1000× magnification was measured and used to generate a frequency distribution. This distribution was then used to calculate the weighted median particle size. The endotoxin content of the particles (in solution at a concentration of 100 µg/mL) was assessed using a chromogenic limulus amebocyte lysate (LAL)-assay (88282; ThermoScientific) according to the manufacturer’s instructions.

### Iron bioavailability

Iron bioavailability (total iron) was quantified in two solutions; Gamble’s solution, to simulate the airway surface lung fluid, and artificial lysosomal fluid (ALF), to simulate the environment when the particles are phagocytosed by macrophages^26^. Particles were prepared at a concentration of 100 µg/mL and left in solution (Gamble’s or ALF) for 24 h prior to assessment of total iron release using a colorimetric iron assay (ab83366; abcam) according to the manufacturer’s instructions. In brief, sample wells were made up to 100 µL with assay buffer. 5 µL of iron reducer was added and plates were incubated at 25 °C for 30 min. 100 µL of iron probe solution was added prior to incubation at 25 °C for 60 min in the dark. Plates were read at OD 593 nm.

### Epithelial cells (A549)

Human alveolar epithelial cells (A549; ATCC) were cultured in Ham’s F-12K medium (21127030; Gibco) supplemented with fetal bovine serum (10%) and glutamine (1%) at 37 °C in humidified 5% CO_2_. Cells were seeded onto plates at 2 × 10^5^ cells/mL prior to exposure to coal particles at concentrations of 0, 50 or 200 µg/mL for 24 h. Relative cytotoxicity was assessed by lactate dehydrogenase (LDH) assay (G1780; Promega) according to the manufacturer’s instructions and expressed as a value relative to control. The production of inflammatory cytokines interleukin (IL)-6 and IL-8 by the cells was quantified in the supernatant by ELISA (R&D Systems) according to the manufacturer’s instructions. Cytokine expression was adjusted to account for variations in cytotoxicity. All experiments were repeated 6 times (fresh cell and coal preparations on different days) to allow statistical comparisons to be made between samples.

### Macrophages (THP-1)

Human monocytes (THP-1; ATCC) were expanded in growth medium (RPMI-1640; ATCC) supplemented with fetal bovine serum (10%) at 37 °C in humidified 5% CO_2_. Cells were seeded onto plates at 2 × 10^5^ cells/mL prior to differentiation into macrophages with (PMA; Sigma-Aldrich) for 48 h. Cells were transferred to PMA-free RPMI for 24 h prior to exposure to coal particles at concentrations of 0, 50 or 200 µg/mL for 24 h. Cytotoxicity was assessed by trypan blue incorporation to assess cell survival and expressed as a value relative to control. The production of inflammatory cytokines tumour necrosis factor (TNF)-α, IL-1β and IL-8 by the cells was quantified in the supernatant by ELISA (R&D Systems) according to the manufacturer’s instructions. Cytokine expression was adjusted to account for variations in cytotoxicity. All experiments were repeated 6 times (fresh cell and coal preparations on different days) to allow statistical comparisons to be made between samples.

### Fibroblasts (CRL-1490)

Fibroblasts (CRL-1490; ATCC) were expanded in Eagle’s Minimum Essential Medium (EMEM; ATCC) supplemented with fetal bovine serum (10%) at 37 °C in humidified 5% CO_2_. Cells were seeded onto plates at 2 × 10^5^ cells/mL prior to exposure to coal particles at concentrations of 0, 50 or 200 µg/mL for 24 h. Cell proliferation was assessed by WST-1 (ab155902; abcam) assay according to the manufacturer’s instructions. The production of collagen by the cells in response to coal particle exposure was assessed by soluble collagen assay (Sircol) according to the manufacturer’s instructions. All experiments were repeated 6 times (fresh cell and coal preparations on different days) to allow statistical comparisons to be made between samples.

### Statistical analysis

Between group comparisons were made using repeated measures analysis of variance (RMANOVA) with Holm-Sidak posthoc tests to assess between group difference when a significant main effect was observed. Where necessary, data were transformed to satisfy the assumptions of homoscedasticity and normal distribution of the error terms. Associations between the response and the coal characteristics (pyrite content and iron release) were assessed by linear regression with adjustment for particle size as a co-variate. P < 0.05 was considered statistically significant; all data are reported as mean (SD).
